# Does the Energy Restriction Intermittent Fasting Diet Alleviate Metabolic Syndrome Biomarkers? A Randomized Controlled Trial

**DOI:** 10.3390/nu12103213

**Published:** 2020-10-21

**Authors:** Yasemin Ergul Kunduraci, Hanefi Ozbek

**Affiliations:** 1 Institute of Health Sciences, Nutrition and Dietetics, Istanbul Medipol University, 34815 Istanbul, Turkey; 2 Department of Nutrition and Dietetics Faculty of Health Sciences, Evliya Celebi Center, Kutahya Health Science University, 43100 Kutahya, Turkey; 3 Department of Pharmacology, Faculty of Medicine, Istanbul Medipol University, 34815 Istanbul, Turkey; hozbek@medipol.edu.tr

**Keywords:** metabolic syndrome, intermittent fasting, energy restriction, weight loss, body composition, blood pressure

## Abstract

The aim of this study was to determine the efficacy of an energy restriction intermittent fasting diet in metabolic biomarkers and weight management among adults with metabolic syndrome. This randomized controlled study was performed with metabolic syndrome patients, aged 18–65 years, at an academic institution in Istanbul, Turkey (*n* = 70). All participants were randomized to the Intermittent Energy Restriction (IER) intervention group and Continuous Energy Restriction (CER) control group. Biochemical tests including lipid profile, fasting plasma glucose, insulin, glycosylated hemoglobin Type A1c (HbA1c), homeostatic model assessment of insulin resistance (HOMA-IR), blood pressure, and body composition were evaluated at baseline and at the 12th week in diet interviews. Dietary intake was measured with the 24-h dietary recall method and dietary quality was evaluated with the Healthy Eating Index-2010. Changes in body weight (≈7% weight loss) and composition were similar in both groups. Blood pressure, total cholesterol, triglyceride, low-density lipoprotein (LDL), fasting glucose, and insulin at the 12th week decreased in both groups (*p* < 0.05). No significant differences were observed in metabolic syndrome biomarkers between the IER and CER groups. The energy-restricted intermittent fasting diet did not cause any deficiencies in macronutrient and fiber intake in the subjects. Healthy Eating Index (HEI) index scores were achieved similarly in both groups, and subjects’ dietary intakes were close to daily reference nutritional intake values. The technique used to achieve energy restriction, whether intermittent or continuous, appears to alleviate the metabolic syndrome biomarkers activated by weight loss.

## 1. Introduction

Metabolic syndrome (MetS), characterized by obesity, hypertension, dyslipidemia, and type 2 diabetes, is a risk factor for cardiovascular diseases [1]. Increased body mass index, visceral adiposity, hyperglycemia, arterial hypertension, and dyslipidemia are accepted as common diagnostic criteria for MetS [2]. Organizations such as the National Heart, Lung, and Blood Institute (NHLBI), the American Heart Association (AHA), the World Health Organization (WHO), the National Cholesterol Education Adult Treatment (NCEP-ATP III), and the International Diabetes Federation (IDF) have proposed diverse diagnostic criteria and cut-off values for MetS [3,4,5]. Depending on the difference in the diagnostic criteria used, the incidence of metabolic syndrome in adults in varied populations has been reported to range from 28 to 50% [6,7,8,9,10]. Turkey, whose adult obesity prevalence is 28.8%, is in the top ten of the adult obesity prevalence ranking in Organization for Economic Cooperation and Development (OECD) countries [11]. Consistent with this high prevalence of obesity, metabolic syndrome prevalence is found to be between 30 and 50% [12,13,14].

In individuals suffering from metabolic syndrome, 5–10% weight loss is recommended for blood glucose regulation, improvement of dyslipidemia, and lowering blood pressure [15]. In order for weight management in obese individuals, energy intake should be less than energy consumption [16]. Energy restriction provides positive effects on metabolic markers such as insulin resistance, blood pressure, and blood lipid profile [17]. One of the alternative ways to create energy restriction is intermittent fasting diets. These diets have a long history and are known to be used in various religious beliefs [18]. Intermittent fasting regimens may benefit from spontaneous calorie restriction when effective on fast days or hours, thereby improving metabolic disorders [19]. Several studies were conducted on fasting diets, such as alternative-day fasting, time-restricted fasting, or Ramadan fasting, in obese individuals. Studies have suggested positive effects on dyslipidemia, hypertension, type 2 diabetes parameters, and cardiometabolic disorders, in addition to providing weight loss [18,19,20,21,22,23]. Preclinical studies and trials have shown intermittent fasting diets—especially time-restricted fasting diets—through incomprehensible mechanisms, have focused on metabolic switching [24]. Time-restricted intermittent fasting methods were a promising strategy for weight loss and improvement of metabolic dysfunctions in a study that compiled several meta-analysis studies on healthy obese or overweight subjects [25]. As far as we know, there is no randomized controlled study evaluating the effects of energy restriction intermittent fasting diets on metabolic syndrome biomarkers in adult patients diagnosed to date. This study aimed to determine the efficacy of an energy restriction intermittent fasting diet in improving metabolic biomarkers and weight management among adults with metabolic syndrome. 

## 2. Materials and Methods 

This study was designed as a randomized controlled clinical trial. All data were collected in accordance with the Republic of Turkey Regulation on Personal Health Research Data (regulation number-date: 30808–21.06.2019) [26]. Subject allocation, diet intervention, and follow-up stages were conducted in accordance with the Ministry of Health Recommended Guideline for the Dieticians (ISBN:978-975-590-659-1-2017) [27]. The study protocol was reviewed and approved by the Clinical Ethics Committee of Medipol University, Istanbul, Turkey (10840098-604.01.01-E.1866-21.06.2019). All participants provided written informed consent before enrolling in the study. The study was registered in clinicaltrial.gov with the number NCT04502329.

### 2.1. Subjects and Recruitment

Based on the assumption that the proposed energy-restricted diet therapies for metabolic syndrome would significantly improve weight management, blood pressure, lipid profile, and glycemic parameters [17], changes in metabolic syndrome components were selected as the primary outcome. As no comparable study has been performed comparing the effects of the 16:8 model of 25% energy restriction diet versus continuous 25% energy restriction diet in diagnosed metabolic syndrome patients, prospective power calculations were not possible. To assess the possibility of type 2 error, retrospective power calculations were conducted according to change weight loss, in which it is known that a 5–10% decrease results in significant improvements in metabolic syndrome components [16]. On the basis of our clinical experience, we hypothesized that the relative improvement in metabolic syndrome components would be greater following weight loss via IER. We calculated that 30 participants per group would provide 80% power to detect a significant difference of 5% in body weight between the intermittent energy restriction fasting group and the only calorie restriction group at the 12th week, using a 2-tailed independent samples *t*-test with α = 0.05. We anticipated a dropout rate of 15%. Thus, we initially aimed to recruit 70 participants (35 per group), assuming that 65 participants (32 IER and 33 CER diet group) would complete the trial. Our dropout rate was less than expected. The sample of the study consisted of all patients with metabolic syndrome who were referred to the diet clinic where the study was conducted after the clinician’s physical assessment. Participants were divided into two groups according to the order of admission to the dietician and allocated 1:1 to treatments using computerized random number generation. The subjects were matched for age and sex before assigning into the groups. Participants were enrolled between June 2019 and January 2020, and dietary interventions and follow-ups were completed in March 2020. The criteria of inclusion were metabolic syndrome patients, aged 18–65 years, with a body mass index (BMI) ≥ 27 kg/m^2^.

Inclusion criteria:

- Being between the ages of 18–65 years;
- Body mass index of 27 and above;
- Referred by a clinician, with a diagnosis of metabolic syndrome according to the criteria of IDF 2005 or NCEP-ATP III.

Exclusion criteria:

- Being pregnant, lactating, pre-menopausal, or menopausal;
- Following a special diet (such as Celiac, type 1 diabetes) or having changed their dietary pattern within the previous 12 weeks of the study’s start date;
- Using a special nutritional supplement (omega-3, probiotic, teff seed, etc.);
- Use of insulin or sulfonamide derivative oral antidiabetic drugs;
- Doing heavy physical activity or working in a physically demanding job;
- Presence of liver or kidney disease, or immune deficiency;
- Conditions that will seriously affect weight management such as having had bariatric surgery;
- Determined to have had an unintentional sudden weight loss of more than 5% in the last three months;
- Intellectual disability or significant medical or psychiatric illness as documented by the referring doctor.

### 2.2. Study Design

Subjects were randomly assigned to one of two groups: intervention, the Intermittent Energy Restriction (IER) group, and control, the Continuous Energy Restriction (CER) group. The first participant was assigned to the IER group to determine dietary feasibility and each subsequent participant was randomly assigned to either the CER group or the IER group. The two groups were randomized using the Microsoft Excel random number generator after being blocked according to sex and age. Participants’ habitual total energy expenditure was calculated with standard method procedures according to the principles followed by the expert consultation guide stated by the World Health Organization (WHO) and Food and Agriculture Organization (FAO). Habitual energy expenditure calculation includes physical activity level and basal metabolic rate. The basal metabolic rate is estimated using Schofield calculations. All participants needed to adhere to a dietary regime, with a reduction of 25% from habitual energy intake for the 12-week intervention period, and maintain their present lifestyle without any change in physical activity levels. Diet menus were prepared considering individual characteristics. IER participants engaged in intermittent fasting. For a 16-h period, such as at 04.00–08.00 a.m., 05.00 p.m.–09.00 a.m., 06.00 p.m.–10.00 a.m., or 07.00 p.m.–11.00 a.m. fasting hours, no food and calorie drinks were consumed. However, participants in fasting hours can drink water, sugar-free tea and mineral water, or black coffee. For the other 8 h, participants followed an energy restriction diet. When preparing the diet menus, a 25% reduction was made on habitual energy consumption. The menu contents were planned appropriately Turkish cuisine and Turkey National Dietary Guidelines which based on the Mediterranean diet. The energy content of the diets was determined at the beginning of the research and was kept at the same level throughout the research. Macro- and micronutrient distribution in the diet was determined according to the personal characteristics of the participants. Meal replacement was explained to individuals with food replica images and verbally. All participants had to buy their own foods. The analysis was carried out at two time points: baseline and end of the 12th week. In order to ensure good compliance, subjects were contacted once a week via telephone, and face to face for four-week periods. Further, all subjects could reach a clinical dietician whenever they wanted. Food diaries and fasting logbooks of the participants were examined during each assessment meeting. Subjects were given detailed instructions verbally and presented with a reference guide on how to fill out the food diaries and fasting log. Adverse events were monitored by clinicians and dieticians during each interview.

The primary outcome of the study was the change in the metabolic syndrome components which were BMI, waist/hip ratio, blood pressure, total cholesterol, low-density lipoprotein cholesterol, high-density lipoprotein cholesterol, triglycerides, fasting glucose, fasting insulin, and insulin resistance by implementing an intermittent energy restriction diet. The potential differences at baseline and the 12th week in anthropometrical measurements such as fat mass (kg), fat mass (%), fat-free mass, total body water, dietary intake, and other biochemical parameters such as ALT, AST, and glycosylated hemoglobin Type A1c (HbA1c) values were all secondary outcomes. All research steps were conducted in accordance with the Turkey Ministry of Health Recommended Guideline for the Dieticians [27].

### 2.3. Body Composition

Height was measured using a standard stadiometer. Body composition was measured using a TANITA SC-330^®^ (TANITA, Tokyo, Japan) body composition analyzer. All measurements were made in the morning during the participants’ fasted state. These instruments were calibrated each time before measurement. Data from these instruments included body weight, body fat percentage, fat mass, fat-free mass, and total body water. Waist circumference was measured by the same dietician with a standard unstretched measuring tape. Body composition was evaluated at baseline and at the 4th, 8th, and 12th weeks in diet interviews. Body mass index (BMI) was calculated as BMI [kg/m^2^] = Weight [kg]/(Height [m])^2^ [28].

### 2.4. Dietary Intake and Physical Activity Level

Dietary intake was investigated with the 24-h dietary recall method in diet interviews by a clinical dietician at baseline, and meetings at weeks 4, 8, and 12. Participants’ daily energy, protein, carbohydrate, fat, fiber intake, and percentage divisions were analyzed with a Nutrition Database Software System BeBIS^®^ (Ebispro for Windows, Stuttgart, Germany; Turkish Version BeBIS, Nutrition Information System, Version 8.1). When the data were unavailable in the software, nutritional information from processed foods was taken from the food label directly. The intake of carbohydrates, fats, proteins, and fiber, and the total energy value were calculated. Dietary intake values which estimated the average of the requirements for the different groups were compared with national dietary guidelines [29]. The intervention and control group subjects’ dietary quality was evaluated with the Healthy Eating Index-2010 in diet interviews. The HEI assessment was based on the US Department of Agriculture’s 2010 index which is revised every five years. This version of the HEI includes 11 components, 9 of which are related to adequate consumption, total fruits, total vegetables, greens and beans, whole grains, dairy products, total protein foods, seafood, plant proteins, and fatty acids. However, 3 focus on restriction refined grains, sodium, empty-calorie groups such as solid fats, and added sugars (SoFAAS). The cut-off points for food consumption or restrictions are expressed in terms of absolute values according to the level of energy consumed by the individual. The HEI scores are classified from 0 to 100, as follows: 80 and above represents a good-quality diet, 51 to 79 a diet requiring improvement, and 51 below a low-quality diet.

The International Physical Activity Questionnaire-Short Form was used to determine the physical activity levels of the participants. According to standard metabolic equivalents corresponding to the duration of the types of physical activity performed at least 10 min at a time in the previous week, the level of physical activity was determined to be sedentary or active. As the level of physical activity was a confounding factor during the diet period, no recommendation was given to the patients in terms of physical activity.

### 2.5. Blood Pressure, Biochemical Markers

Systolic and diastolic blood pressures were measured two times with four minute intervals in each time by an automatic oscillometric device (Omron M2 Basic, Kyoto, Japan) after participants had rested in a seated position for 5 min. Blood pressures was evaluated at baseline and the 12th week in diet interviews. All participants visited their clinicians before the dietary intervention and after 12 weeks. Biochemical test results were obtained from participants’ clinicians at baseline and week 12. All samples were collected by trained personnel using standard operating procedures and analyzed in the same laboratory. Biochemical markers, including blood lipids (total cholesterol, triglycerides, high-density lipoprotein (HDL), low-density lipoprotein (LDL)), fasting plasma glucose, and Haemoglobin A1c (HbA1c) were obtained by standard methods. Serum concentrations of insulin were measured by immunonephelometric methods. Insulin resistance was estimated with the homeostasis model assessment (HOMA-IR) and calculated as HOMA-IR = (Fasting plasma glucose [mg/dL] × Serum insulin [IU/L])/405 [30].

### 2.6. Statistical Analysis

Data were analyzed using Statistical Package for the Social Sciences (SPSS), version 20.0 for Windows (SPSS Inc., Chicago, IL, USA). Tables and figures were created using Microsoft Word and Excel 2010 for Windows (Microsoft Inc., North Bergen, NJ, USA). Results are expressed as the mean ± standard error of the mean (SEM) 95% confidence interval. Normality tests were evaluated using the Shapiro–Wilk test. Initially, baseline characteristics were measured, in which parametric ordinal data differences between two groups were measured using independent samples *t*-tests. Nominal data were compared with chi-square tests. Basically, the parametric measurements of each participant in the same group at baseline and the 12th week were compared with the paired *t*-test. Nonparametric measurements of each participant in the same group at baseline and the 12th week were compared with the Wilcoxon rank sum test. Considering the two groups were not balanced with respect to baseline body weight, the results of changes in body weight were reported in both absolute (kg) and relative (%) terms. In addition, at the end of the 12th week, parametric ordinal measurements between the two groups were compared using the independent samples *t*-test, whereas nonparametric measurements were compared with the Mann–Whitney U test. A *p*-value of < 0.05 was considered statistically significant.

## 3. Results

### 3.1. Study Participants

**Figure 1:**
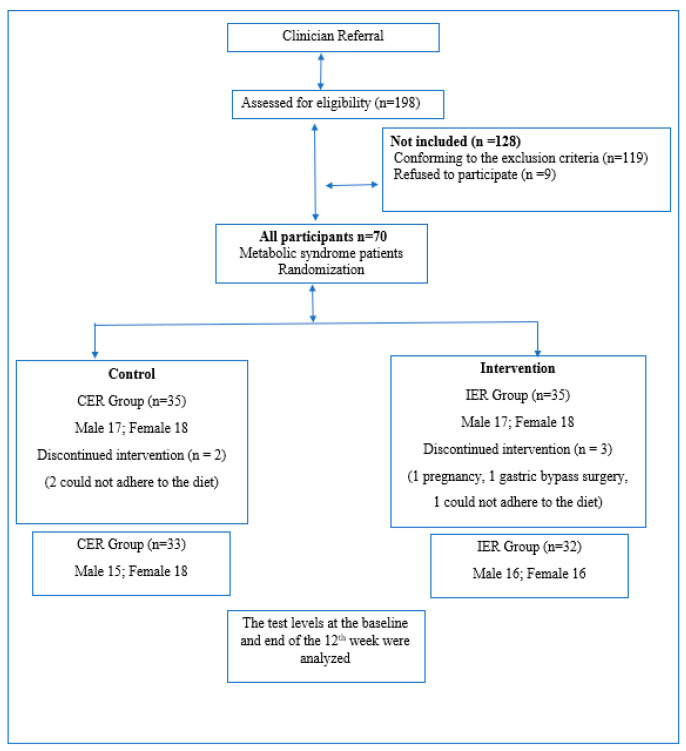
Study flow chart. IER, Intermittent Energy Restriction; CER, Continuous Energy Restriction.

Seventy subjects met all the criteria and entered the study. Participants were randomized to IER or CER groups (*n* = 35) equally. Five participants did not complete the whole protocol. One dropped out due to pregnancy (IER group), three did not adhere to the diet (one from the IER group, two from the CER group), and one withdrew because of elective gastric bypass surgery (IER group). Sixty-five subjects completed the whole study seamlessly without encountering any side effects (see Figure 1).

The baseline characteristics of participants who completed the trial are shown in Table 1. There were no significant differences in baseline characteristics between IER and CER groups except BMI.

### 3.2. Body Composition

The IER group lost a significant amount of weight loss during the 12-week study period (about 8%, *p* < 0.001). Further, the CER group lost weight and the changes significantly occurred (about 6%, *p* < 0.001). In the 12-weeks measurements, there is a significant difference between the body weight means of the IER and CER groups. While a decrease of approximately 5.5 kg was observed in the amount of body fat in the IER group, a decrease of approximately 4 kg was observed in the CER group. Although the participants had different diets, improvements were observed in body fat percentage, lean body mass, total body water, body mass index, and waist/hip ratio during the diet (see Table 2).

### 3.3. Blood Pressure, Lipid Profile, Glycemic Measures

At week 12, systolic and diastolic blood pressure, LDL, TC, TG, fasting glucose, HOMA-IR, and HbA1c decreased significantly in both groups. Insulin decreased only in the CER group significantly. However, there were no inter-group differences in blood pressure, lipid profile, and glycemic parameter changes. HDL values remained almost similar in both IER and CER groups (see Table 3).

### 3.4. Dietary Intake

There were no significant differences in nutritional habits like daily meal skipping/or not, and which meal was skipped between the groups at baseline (Table 1). IER and CER groups also had a similarly decreased energy intake of about 500 kcal/day. The intake of carbohydrates, fats, and proteins showed a significant decrease in both groups at the end of the diet compared to the beginning. However, dietary compositions are similar in both groups from baseline to end of the 12th week in terms of the percentages of energy from macronutrients. Dietary fiber consumption in the CER group had a significant decrease, but there was no significant difference between the two groups. Diet composition was also similar from the baseline to the end of the 12th week. Compared to reference values, it is observed that energy, protein percentage, and fiber intake levels in the 12th week are similar, and carbohydrates and fat consumption remain slightly above the recommended values (Table 4).

**Figure 2:**
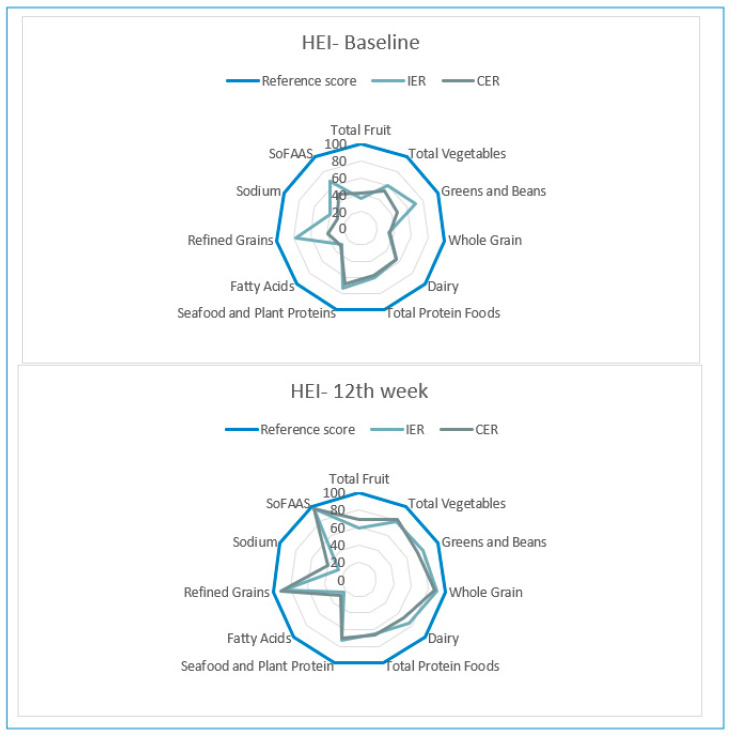
Healthy Eating Index (HEI-2010) components radar graphs for IER and CER groups (baseline–12th week).

According to baseline HEI scores, diet quality was low, but also at the 12th week, IER and CER group participants were classified as requiring improvements in diet quality. HEI scores were similar at baseline and the 12th week in both groups (Table 4). After the 12-week diet, the participants showed similar progress in increasing the consumption of recommended foods and restricting foods that should be restricted, except for sodium and fatty acids. Although the diets employed in both study groups are different, it is observed that HEI components have similar scores (Figure 2).

## 4. Discussion

This randomized controlled trial investigated for the first time the clinical effects of 12 weeks of intermittent fasting therapy in patients with metabolic syndrome. The intervention of intermittent energy restriction caused a mean 8% weight loss, reduced waist/hip ratio, and was not associated with any adverse effects. While intervention and control groups did not differ significantly in blood pressure, lipid profile, and glycemic measures, all participants had positive progress in all metabolic parameters except HDL. However, the sample size in this study appeared to result in some underpowering for outcomes. Our findings indicate that energy-restricted diets led to significant improvement in all cases who suffered from metabolic syndrome. Compared to the continuous energy-restricted diet, the intermittent energy restriction diet was not significantly superior in terms of changes in patients’ blood pressure, lipid profile, and glycemic measurements.

There has been limited research into IER in patients suffering from metabolic syndrome, and the majority concerns obesity or overweight subjects. According to a study in which there was intermittent fasting versus continuous energy intake at 100% or 70% of requirements on insulin sensitivity, cardiometabolic risk, and body composition in metabolic risk in women with overweight, a 70% energy restriction fasting diet displayed greater reductions in fat mass, weight, cholesterol, and LDL values compared with continuous 70% energy restriction or eucaloric fasting [31]. Chow et al. (2019) investigated the effects of a 12-week time-restricted diet (ad libitum intake during an 8-h eating window) on body composition and metabolic measure in subjects who are overweight and obese. Compared with the non-time-restricted diet intervention, the time-restricted diet reduced weight, lean mass, and visceral fat (*p* ≤ 0.05). Blood pressure, glycemic measure as HbA1c %, HOMA-IR, fasting glucose, and fasting insulin values were similar in both groups at the 12th week [32]. In a randomized controlled short-term fasting diet study conducted on male and female participants with central obesity, the intervention group diet monitored a daily 600 kcal energy intake twice a week, followed by five-day healthy eating advice. The control group participants monitored a daily 500 kcal energy restriction diet with healthy eating advice. According to the results of this four-week study investigating the effects of these different diets on cardiometabolic mechanisms, improvements were found in metabolic parameters such as insulin resistance, blood pressure, and lipids in both groups, regardless of weekly energy distribution [33]. A systematic review of a meta-analysis about intermittent energy restriction on obese or overweight patients confirms that intermittent energy restriction has no evidence of being superior to continuous energy restriction for weight loss [34]. The probable reason for the inconsistent finding of the current study is our study design discrepancy. Both diet groups in this study similarly performed a 25% energy restriction of habitual energy intake, not the ad libitum or non-energy-restricted diet like other studies.

Another study investigated the effect of early time-restricted feeding on blood pressure and insulin sensitivity in men with pre-diabetes. When the five-week randomized crossover isocaloric and eucaloric time-restricted diet models were tested, it was found that the early time-restricted diet had a positive effect on insulin levels and blood pressure. The difference between the early time-restricted diet and other intermittent fasting diet interventions was that the period was between 06:30 and 08:30 for breakfast and the last meal was at 15:00. It has been reported that this type of practice in intermittent fasting types is promising in terms of cardiometabolic health due to its compatibility with the human circadian rhythm [35]. The dual-energy X-ray absorptiometry method has been used in many studies investigating the effectiveness of intermittent fasting diets in body weight management [31,32]. In this study, the bioelectric impedance method was used. In a study comparing the adequacy of these two methods in measuring body composition in adults with chronic heart disease, it was reported that the dual-energy X-ray absorptiometry method calculated lean body mass to be slightly more than it was, and BIA (Bioelectrical Impedance Analysis) was insufficient [36]. On the other hand, in a Japanese study, the authors showed that measurement of the visceral fat level by BIA is useful for the detection of MetS because it is correlated with all metabolic parameters [37]. Further, Bintvihok et al. used the bioelectrical impedance analyzer TANITA SC-330^®^ (TANITA, Tokyo, Japan); this device was useful for screening MetS in women with MetS in Thailand [38]. In order to determine the effects of intermittent fasting diets on body weight and composition, it will be useful to investigate different methods with high reliability in the field.

The IER diet did not negatively impact an adequate and balanced nutrition. Daily food consumption records show that there was no significant difference in total energy, carbohydrates, protein, fat, and fiber intake between the two diets at baseline or at the 12th week, which helps to explain why the score of HEI in the two groups was close. Essentially, in each group, the degree of energy restriction was the same; only the strategy to achieve that restriction differed. The only difference between the two diet types is the limitation of 8 h of eating hours and 16 h of fasting. Previous research has shown that only a time-limited diet without calorie counting is also effective for weight management [31]. Both groups succeeded in meeting the recommended average intakes of fiber in 12 weeks (25 g/day for adults). According to the Turkish National Nutrition Survey 2010, the major source of fiber in the standard Turkish diet is grains, cereal foods, fruits, and starchy vegetables [39]. This is a problem for weight management as the national average intake of these foods is well above the current recommended level. As can be seen from the HEI scores, the score from refined grains approached the reference scores by dietary intervention, since whole grain consumption is recommended for all patients. So fiber intake reached desirable levels despite energy restriction.

There are some limitations to the current study: namely, randomization was stratified by sex and age only and resulted in a significant baseline higher mean body weight and body mass index in the CER group because of the small number of the sample size. In other studies, to be carried out in the future, selecting according to body mass index during randomization will be more effective in comparing the weight loss of subjects using different diets. Another limitation is that macronutrient composition of the diet was based on patient interviews. Due to the limitations, nutrient intake could have existed and played a role in the accuracy of the observed outcomes. In our study, the 16:8 model of intermittent fasting diet fasting/feeding times planned four alternate periods because of increased adherence to the diet. Human is a biopsychosocial being, so from this point of view, flexible periods of fasting diets appear to be a feasible and sustainable option. Although the intervention was completed with different start and end times compatible with the circadian rhythm, the fact that the regimen could not be performed in a fixed time interval for all participants is the weakness of this study.

## 5. Conclusions

The results of the study indicate that an energy restriction intermittent fasting diet is a feasible weight loss strategy to improve metabolic syndrome and it is well tolerated. Moreover, the diet does not appear to cause an unbalanced nutritional intake. Larger randomized trials with longer observation periods should test the clinical effectiveness of fasting programs in metabolic syndrome patients.

## Figures and Tables

**Table 1:** Baseline characteristics of metabolic syndrome patients who completed the trial.

Characteristics	IER Group (*n* = 32)	CER Group (*n* = 33)	*p*-Value
Age (years)	47.44 ± 2.17	48.76 ± 2.13	0.718
Gender ratio (men/women)	16:16	15:18	0.714
BMI (kg/m^2^)	36.58 ± 0.93	32.82 ± 0.72	0.002
Fat mass (%)	39.53 ± 1.23	37.06 ± 1.34	0.180
Waist to hip ratio	1.05 ± 0.02	1.04 ± 0.02	0.747
Physical activity ratio (sedentary/active)	23:9	27:6	0.381
Daily meal skipping/or not	25/7	26/7	0.948
Skipped meal (breakfast/lunch/dinner)	9:14:2	8:16:2	0.768
Fasting glucose	119.19 ± 7.63	115.06 ± 5.96	0.494
Triglycerides	212.31 ± 23.52	197.61 ± 29.95	0.168
HDL cholesterol	42.50 ± 1.77	46.65 ± 2.24	0.152
TSH (mU/L)	2.16 ± 0.27	1.98 ± 0.22	0.788

Data presented as mean ± SEM. IER: İntermittent Energy Restriction; CER: Continuous Energy Restriction; BMI: body mass index; HDL: high-density lipoprotein; TSH: thyroid stimulating hormone. *p*-values for comparison between groups at baseline.

**Table 2:** Body composition measurements in both groups.

	IER Group (*n* = 32)			CER Group (*n* = 33)			*p*-Value **
	Baseline	12th Week	*p*-Value *	Baseline	12th Week	*p*-Value *	
Weight	97.53 ± 2.82	89.26 ± 2.41	<0.001	88.43 ± 2.00	82.62 ± 1.76	<0.001	0.029
FM (kg)	38.79 ± 1.80	33.27 ± 1.59	<0.001	32.89 ± 1.56	28.80 ± 1.51	<0.001	0.045
FM (%)	39.53 ± 1.23	37.10 ± 1.35	<0.001	37.06 ± 1.34	34.61 ± 1.40	<0.001	0.207
FFM (kg)	58.73 ± 1.85	55.98 ± 1.80	<0.001	55.54 ± 1.59	53.83 ± 1.44	<0.001	0.352
TBW (kg)	43.43 ± 1.34	40.80 ± 1.33	<0.001	40.77 ± 1.06	38.98 ± 1.02	<0.001	0.281
BMI (kg/m^2^)	36.58 ± 0.93	33.52 ± 0.87	<0.001	32.82 ± 0.72	30.69 ± 0.65	<0.001	0.011
W/H	1.05 ± 0.02	1.01 ± 0.02	<0.001	1.04 ± 0.02	1.00 ± 0.01	<0.001	0.904
TWL		8.27 ± 0.81			5.80 ± 0.65		0.020
TWL (%)		8.32 ± 0.64			6.42 ± 0.64		0.041
TTW (cm)		6.84 ± 0.57			5.15 ± 0.55		0.015

Data presented as mean ± SEM. IER: İntermittent Energy Restriction; CER: Continuous Energy Restriction; FM: fat mass; FFM: fat-free mass; TBW: total body water; BMI: body mass index; W/H: waist/hip ratio; TWL: total weight loss; TTW: total thinning around waist circumference; * *p*-values are for changes between time points within groups. ** *p*-values are only for comparisons between the two groups’ measurements at the end of the 12th week.

**Table 3:** Changes in blood pressure, lipid profile, and glycemic measures in the IER and CER groups.

	IER Group			CER Group			*p*-Value **
	Baseline	12th Week	*p*-Value *	Baseline	12th Week	*p*-Value *	
SBP (mm Hg)	131.88 ± 2.49	124.53 ± 2.11	<0.001	140.73 ± 2.69	127.73 ± 1.85	<0.001	0.146
DBP (mm Hg)	83.97 ± 1.36	79.22 ± 1.15	<0.001	89.06 ± 1.66	80.85 ± 0.95	<0.001	0.277
HDL (mg/dL)	42.50 ± 1.77	43.03 ± 1.78	0.173	46.65 ± 2.24	46.27 ± 2.10	0.175	0.244
LDL (mg/dL)	147.19 ± 5.96	130.19 ± 4.80	<0.001	148.12 ± 5.80	132.15 ± 4.28	<0.001	0.761
TC (mg/dL)	226.88 ± 8.14	197.56 ± 6.58	<0.001	230.09 ± 8.66	200.73 ± 6.15	<0.001	0.726
TG (mg/dL)	212.31 ± 23.52	170.47 ± 12.60	<0.001	197.61 ± 29.95	157.61 ± 13.53	<0.001	0.362
Glucose (mg/dL)	119.19 ± 7.63	103.72 ± 2.70	<0.001	115.06 ± 5.97	101.94 ± 2.40	<0.001	0.777
Insulin (IU/L)	14.40 ± 2.69	12.17 ± 1.81	0.118	15.81 ± 0.34	13.42 ± 1.57	0.046	0.462
HOMA-IR	4.88 ± 0.74	3.59 ± 0.50	<0.001	4.09 ± 0.80	3.15 ± 0.51	0.004	0.369
HbA1c (%)	6.56 ± 0.31	6.24 ± 0.26	<0.001	6.41 ± 0.25	6.10 ± 0.16	<0.001	0.777

Data presented as mean ± SEM. IER: İntermittent Energy Restriction; CER: Continuous Energy Restriction; SBP; systolic blood pressure; DBP: diastolic blood pressure; HDL: high-density lipoprotein; LDL: low-density lipoprotein; TC: total cholesterol; TG: triglycerides; Glucose: fasting glucose; HOMA-IR: homeostatic model assessment of insulin resistance; HbA1c: glycosylated hemoglobin Type A1c. * *p*-values are for changes between time points within groups. ** *p*-values are only for comparisons between the two groups’ measurements at the end of the 12th week.

**Table 4:** Changes in energy, carbohydrate, protein, fat, dietary intakes, and Healthy Eating Index (HEI) scores in the IER and CER groups.

	IER Group (*n* = 32)			CER Group (*n* = 33)			*p* Value **	References Values ^a^
	Baseline	12th Week	*p*-Value *	Baseline	12th Week	*p*-Value *		
Energy (kcal)	2066.14 ± 71.13	1496.54 ± 60.42	<0.001	2043.50 ± 61.87	1519.52 ± 51.52	<0.001	0.773	M: 2200 kcal; F:1600 kcal
CH (g)	201.99 ± 8.55	151.58 ± 7.67	<0.001	216.10 ± 10.90	158.46 ± 5.10	<0.001	0.458	130 g
CH (%)	39.23 ± 1.24	40.66 ± 1.51	0.451	42.10 ± 1.48	42.32 ± 1.13	0.893	0.38	45–60%
Protein (g)	85.63 ± 3.94	67.66 ± 3.49	<0.001	80.54 ± 3.85	72.62 ± 3.94	0.038	0.351	M: 63.1 g; F: 55.2 g
Protein (%)	16.66 ± 0.57	18.25 ± 0.73	0.095	15.96 ± 0.53	19.13 ± 0.80	<0.001	0.423	M: 10–20; F: 14–20%
Fat (g)	98.89 ± 5.28	66.16 ± 4.21	<0.001	92.71 ± 4.31	63.26 ± 3.66	<0.001	0.603	M: 67.2 g; F: 48.8 g
Fat (%)	42.82 ± 1.49	39.43 ± 1.52	0.112	41.11 ± 1.58	36.8 ± 1.49	0.045	0.221	20–35%
Fiber (g)	22.68 ± 1.32	24.86 ± 1.50	0.237	22.77 ± 0.98	27.92 ± 1.22	0.001	0.118	25 g
HEI score	53.69 ± 2.84	70.66 ± 1.82	<0.001	44.06 ± 3.25	72.33 ± 1.79	<0.001	0.513	0–100

Data presented as mean ± SEM. IER: İntermittent Energy Restriction; CER: Continuous Energy Restriction; CH: carbohydrate; M: Male; F: Female. ^a^ reference values of energy, carbohydrates, proteins, fats, and fiber are compared with the reference values of the Turkey National Dietary Guidelines 2016. HEI scores mean; ≤51: low quality; 51–79: requiring improvement; ≥80: good-quality diet. * *p*-values are for changes between time points within groups. ** *p*-values are only for comparisons between the two groups’ measurements at the end of the 12th week.
